# Supplementary material for: Muscle growth and anabolism in intensive care survivors (GAINS 2.0): Protocol for a multi-centre randomised; placebo controlled clinical trial of nandrolone in deconditioned adults recovering from critical illness
Source: PLoS One. 2025 Feb 20;20(2):e0315170. doi: 10.1371/journal.pone.0315170 (PMC11841879; doi:10.1371/journal.pone.0315170)
Supplement: S1 File — (PDF) [file pone.0315170.s002.pdf]

CLINICAL TRIAL PROTOCOL

Muscle growth and anabolism in intensive care survivors

(GAINS 2.0)

A multi-centre randomised, placebo controlled clinical trial of nandrolone in deconditioned adults recovering from critical illness

Protocol version 1.5
Date 13/03/2024

Trial registration: ANZCTR : ACTRN12616000835448

Ethics approval : TBA : RGS0000004839

Administrative information:

Chief investigator

CPI A/Prof Matthew Anstey

Specialist in Intensive Care Medicine,

Sir Charles Gairdner Hospital

+61409124876

Matthew.anstey@health.wa.gov.au

Investigators :

PI FSH : A/Prof Ed Litton

PI RPH Dr Julian Sunario

PI SCGH: Dr Brad Wibrow

AI: Prof KM Ho

AI Robert Palmer

AI Nat Tran

AI Bianca Mammana

Role of the funders

This is an investigator-initiated study. The management committee will take responsibility for study design and oversight. The funders will have no role in study design, data collection, management, analysis, data interpretation, manuscript writing, or in the decision to submit manuscripts for publication.

Coordinating centre and data management centre

Sir Charles Gairdner Hospital acts as the co-ordinating centre and data management centre:

1. INTRODUCTION

1.1 Background and rationale

Intensive care patients can face significant health issues that extend beyond their Intensive Care Unit (ICU) stay. Despite recent advances it is estimated that one-quarter to one-half of long-stay intensive care survivors live with significant weakness as a consequence of their illness, resulting in impaired mobility and function.(1,2)

The loss of muscle mass in critical illness is related to immobility and a complicated process that causes muscle and nerve dysfunction called critical illness polymyoneuropathy. Another contributory factor is low levels of anabolic (muscle building) hormones such as testosterone – with testosterone levels in critically ill patients are extremely low, even in the recovery phase from acute illness. (3,4) One potential treatment may be to provide anabolic support in the recovery phase from prolonged critical illness.

This project aims to test whether giving a synthetic testosterone (nandrolone), will improve muscle strength in ICU survivors, when compared to placebo. Previous research has already established that early physiotherapy in the ICU can reduce length of stay and improve patients outcomes. In this study, both groups will receive standard care, which includes early physiotherapy. Nandrolone or placebo will be administered intramuscularly weekly for up to 3 weeks. Outcome measures will include hospital length of stay, time until the patient walks with assistance, muscle strength (globally and grip strength) as well as the patient's physical functioning at 3 months following enrolment.

The study design will be a double blinded randomised controlled trial, that will involve patients in WA Intensive Care Units (4 of the major units have already expressed interest in participating) and well as interstate if required.

The teams involved are multi-disciplinary, involving physiotherapy, dietitians, pharmacy as well as medical specialists. The investigators have already successfully conducted a pilot feasibility trial of a nandrolone versus placebo, showing that the intervention is safe and feasible.(5)

Given the large and increasing numbers of patients surviving intensive care both locally and internationally, (as illustrated recently with the post COVID syndrome) but with physical impairments, the results of this study would be of great interest to clinicians and patients alike.

Current recommendations for anabolic steroids in critical illness

Not part of standard care.

1.2 Objectives

Aims

1. To reduce the time until a patient walks with one assist through the addition of an anabolic steroid (nandrolone) and resistance exercise to deconditioned ICU patients.
2. To determine whether nandrolone reduces hospital length of stay for deconditioned ICU patients.
3. To determine whether nandrolone reduces the time to return to work for deconditioned ICU patients (of working age).
4. To document changes in grip strength and limb muscle strength in the two groups in the follow-up period after enrolment.

HYPOTHESES:

Nandrolone will improve muscle strength and physical functioning at hospital discharge and at follow-up 3 months after enrolment.

1.3 Trial design

This study is a multicentre, parallel group, placebo-controlled, randomised, superiority trial which will allocate ICU patients in a 1:1 ratio to nandrolone in addition to standard care compared with placebo in addition to standard care.

2. METHODS: PARTICIPANTS, INTERVENTIONS, AND OUTCOMES

2.1 Study setting

This study will be conducted in Intensive Care Unit (ICU) and High Dependency Units (HDU) of several hospitals in Western Australia. For the purposes of this study, an HDU admission is defined as an admission of a critically unwell patient to a hospital area that includes specialised nursing at a ratio of 1:2 or 1:1.

2.2 Eligibility criteria

2.2.1 Inclusion criteria

1.) At least 21 years of age
2.) Admitted to participating ICU/HDU
3.) Receiving nutrition at estimated goals for at least 24 hours
4.) ICU/HDU length of stay => 5 days
OR
Significant weakness as deemed by treating clinician below patient's baseline as a result of the ICU stay

2.2.2 Exclusion criteria

1.) Intercurrent septic shock (fevers, broad spectrum anti-microbials, and needing vasopressors)
2. Active cardiac disease (such as STEMI/NSTEMI in last 2 weeks) or EF <35%
3. Prostate or breast cancer
4. Ongoing reason for catabolic state (active malignancy, HIV & opportunistic infection last 2 months)

5. Unable to engage in rehabilitation (due to significant neurological or orthopaedic issues)
6. Normal age-related level of serum testosterone in males (measured in early morning 6-9am)
7. Pregnancy or breast-feeding
8. Any known allergies to nandrolone components – including peanuts and soya and latex.
9. Elevated LFTs (ALT > 5x normal) and impaired bilirubin excretion
10. Polycythaemia (Hb > 165 males, > 150 females)
11. Nephrotic syndrome.
12. Athletes competing in international/national events.
13. Expected death within the next week

2.3 Interventions

2.3.1 Study interventions

Intervention

Standard care (see below) with the addition of IMI nandrolone.
Dosing: Males: 100 mg weekly. Females: 50mg weekly – for 3 weeks or until hospital discharge, whichever is earlier.
The study drug will be drawn up by the ICU pharmacist, and then the syringe will be covered (to mask the contents of the syringe). All investigators, clinicians, nursing staff and patient are blinded.

NOTE: Dose change from the pilot study. The dosing was 200mg males/ 100mg females – however this is a large intramuscular volume, and can be difficult in patients without much muscle mass.

Comparator group

Will receive : standard care consists of usual physiotherapy interventions, which includes early mobilisation by staff. Early mobilisation in the ICU follows a graduated increase in activity, from passive range of motion in bed to active motion in bed to sitting/balance to assisted activity and then independent activity.
Placebo = sterile water injection or
Placebo = Normal saline (Nacl 0.9 %)

Concomitant therapies

We will record

Physiotherapy received - Duration of rehabilitation physiotherapy and mobility achieved (ventilation physiotherapy treatments will not be counted).
Nutrition received - Caloric intake (kcal/total), Protein intake (g/total), Food intake (% of meals, and extrapolated calories based on meal charts from hospital).

Outcomes

Primary and secondary outcomes

Primary outcome:

1. Time to walking with one person assisting (days from admission to ICU).
2. Hospital length of stay

Secondary outcomes:

1. Change in muscle strength as measured by the medical research council muscle strength (MRC) sum score - enrolment to discharge (note some ICU patients may not be able to participate in full MRC scoring, score will be extrapolated from available limbs) (9)
2. Grip strength (measured by hand held dynamometry)

3. Functional activity at hospital discharge (measured by physiotherapy staff using the Chelsea critical care physical assessment tool (CPAx (7)) and follow-up phone call at 3 months post discharge (using SF-36).
4. Hospital length of stay
5. Time to return to work (days) (for those working pre ICU).

1.1 Statistical analysis and Sample size

Trial will be prospectively registered on ANZCTR.

Efficacy of intervention will be analysed on an intention to treat basis. The analysis population is the intention to treat population defined as all study participants except for those who do not consent to use of data. There will be no imputation for missing data. A per protocol analysis will also be conducted including all participants with adherence to study medication of >80% of total study duration.

Normally and non-normally distributed data will be presented as mean and standard deviation (SD), and median and interquartile range (IQR), respectively. Between-group comparison of parametric data will be provided as mean difference and confidence interval and analysed using Student's t test. Between-group comparison of non-parametric data will be presented at median difference and analysed using the Mann-Whitney U test. For dichotomous data, frequencies and percentages will be presented and between-group analysis will use Fischer's Exact or Chi-squared test as appropriate. The numbers at risk in each group and the number and proportion of events observed will be reported, as well as the equivalent absolute risk difference, relative risk ratio and corresponding 95% confidence intervals. Survival time from randomisation until day 60, according to treatment group will be displayed as Kaplan-Meier curves and analysed using a log-rank test. Estimates of hazard ratios for survival, with corresponding 95% CI and P values, will be obtained from the Cox proportional hazards models incorporating treatment group alone, and independent covariates used in the multivariate models. Secondary analysis will also be conducted adjusting the primary outcome variable for prespecified baseline covariates (age, gender, APACHE-II score, BMI) in a transformed multivariable linear regression model including

baseline univariate variables with a $p<0.25$. For all outcome analyses a two-sided P value of <0.05 will be considered significant.
Baseline variables recorded to demonstrate that the groups are comparable; if this is not demonstrated, *post-hoc* adjustments to correct for differences will be applied.
Sample size calculation: 60.
This is based on several different assumptions and outcome measures:

1. Hospital length of stay, as sample of n=24 per group has 80% power to detect a median difference of 3 days LOS (26 vs 23 days) with IQR 5.4 between independent groups (this is a conservative estimate as in the pilot, LOS results were 26 vs 36 days).
2. Time to walking with one-assist (ICU mobility scale). A sample of 24 per group has 80% power to detect a difference in proportions of 40 between the intervention and control groups, of patients walking with one assist at discharge (from a baseline of 27% in pilot).

To allow for loss to follow-up, the sample size number increased to 60.
Subgroup analysis: due to potential heterogeneity in patients, stratification based on MRC score, >= 48 (48 is an accepted cut off for ICU acquired weakness).

Economic evaluation:
An economic evaluation will be performed. Cost offsets from any reduction in LoS will be estimated separately for ICU stay and total hospital stay, and costed using hospital-specific per day costs. We will estimate QALYs using the SF-36 data converted to SF-6D using Australian population weights.(6)

METHODS: ASSIGNMENT OF INTERVENTIONS

Sequence generation

A permuted block randomisation method will be used with variable block sizes, stratified by site.

Allocation concealment

Allocation concealment will be maintained by using sealed, sequentially numbered, opaque envelopes at each study site containing the study number corresponding to the allocated study number. Randomisation will not be performed until participants fulfil all eligibility criteria and are ready to be assigned to study treatment.

Implementation

Participants will be enrolled in the study by ICU doctors, and research staff and study enrolment will be communicated to the bedside nurse who will implement the study intervention. Study drug will be prescribed on the medication chart as 'GAINS STUDY DRUG : NANDROLONE OR PLACEBO IMI"

Blinding

Study medication will be provided blinded to treatment providers and trial participants through the use of a placebo. Syringes will be masked (sticker/alfoil) to prevent the contents being visualised to the administering nurse. Outcome assessment at 3 months, including SF-36, return to work will be conducted by a blinded research team member via telephone.

DATA COLLECTION, MANAGEMENT, AND ANALYSIS

Data collection methods

Data Collection

Data will be collected on a prespecified eCRF (RedCap - Research Electronic Data Capture - a secure web application for building and managing online surveys and databases) supported by a study-specific data dictionary and including checks of logical consistency and automatic query generation.

The following data will be collected:

- Age
- Gender
- ANZICS APD number

- Admission type (elective vs. emergency)
- ICU admission source (i.e. Emergency Department vs. ward vs. theatre vs. other hospital)
- Chronic APACHE co-morbidities
- APACHE-III admission diagnosis
- Illness severity based on the on the APACHE-II and III scores and risk of death, and "ANZ Risk of Death" models score
- ICU or HDA mortality
- ICU or HDA LOS
- Total duration of mechanical ventilation
- Total duration of vasoactive medication requirement
- Incidence and duration of renal replacement therapy
- Hospital mortality
- Post-ICU ward LOS
- Hospital disposition (home vs other acute care facility vs rehabilitation)
- ICU readmission
- Hospital readmission
- Day 60 mortality
- Inflammatory markers (CRP, Procalcitonin and WBC count at enrolment, ICU discharge and hospital discharge)
- BMI, weight at hospital admission, pre-admission medications.
- Baseline SF-36, FIM score and Barthel Index, to determine the patient's pre-existing functional status (based on status two weeks prior to admission to ICU, from patient or surrogate).

- **Measurement of variables:**

- **1. Laboratory tests**

- Pregnancy test in women of child bearing age
- Baseline: sex hormone binding globulin, testosterone.

- Routine blood tests will occur as planned by treating clinicians, and will collect:
- Haemoglobin, white cell count, albumin, urea and creatinine, alanine transferase.
- Lipids at baseline and at day 15-21.
- **2. Strength and weight measurements**
- a) Weekly: Hand grip strength, weight (kg) and MRC.
- **3. Other measures**
- Physiotherapy received - Duration of rehabilitation physiotherapy and mobility achieved (ventilation physiotherapy treatments will not be counted).
- Nutrition received - Caloric intake (kcal/total), Protein intake (g/total), Food intake (% of meals).

- **Follow-up**
- Phone call follow-up at 3 & 6 months using the SF-36 score , and asking about return to work (see script page).

Pre-specified Subgroups:

BMI at enrolment < 20

Admission cause :sepsis, neurological, post surgical.

Data will be collected by trained research co-ordinators at each site. Patients and/or their next of kin will be asked to provide three possible points of contact (home and close family contact details) to the research staff prior to discharge. Day 90& 180 (post hospital discharge) SF-36 will be conducted over the phone by a trained assessor.

For data collected by research co-ordinators at each site, data quality and protocol standardisation will be optimised by arranging a start-up meeting, providing an early on-site monitoring visit, regular feedback to each centre via phone and the trial web-site, and a monthly newsletter. A complete study procedures manual will be produced. All study personnel will have 24 hour access to the study coordinating centre to resolve any questions.

1.2 Data management

Study data will be entered directly into a secure web-based case report form with the option to print blank and completed forms. A detailed study monitoring plan will be prepared by the study management committee prior to commencement of the study. On site source monitoring will be conducted by the co-ordinating centre and will include 100% source data verification for the primary end point. Source data verification will be completed for all data points for the first two patients at each centre and for a random sample of patients during monitoring visits thereafter.

1.3 Records retention

Once the study is completed, paper records will be shredded and electronic database will be destroyed 15 years after the completion of the study. Only authorised study personnel will have access to the electronic database. At the time of study completion, computer records will be copied to a compact disc for long term storage and back up.

METHODS: MONITORING

Data monitoring

Data Monitoring Committee composition and governance

An independent Data Monitoring Committee (DMC), consisting of experts in intensive care and rehabilitation will be established before patient enrolment.

Interim analyses

No interim analysis is planned. However, all SAEs will be provided to the DSMB and the DSMB reserves the right to call for a blinded or unblinded interim analysis at any point in the conduct of the trial.

Harms

It is recognised that the intensive care patient population will experience a number of common aberrations in laboratory values, signs and symptoms due to the severity of the

underlying disease and the impact of standard therapies. Intensive care patients will frequently develop life-threatening organ failure(s) unrelated to study interventions and despite optimal management. Therefore, consistent with established practice in academic ICU trials[34], events that are part of the natural history of the primary disease process or expected complications of critical illness will not automatically be reported as serious adverse events in this study. All adverse events which are considered to be potentially causally related to the study intervention or are otherwise of concern in the investigator's judgement will be reported.

Potential side effects: Cardiac failure, Myocardial ischaemia, Virilisation in females (hoarseness, acne, hirsutism), abnormal LFTs, Polycythaemia, Increased sensitivity to oral anticoagulants.

Safety endpoints include: new onset hypertension (SBP>180units), abnormal liver function tests (ALT increase > 100% from baseline and >100), cardiac ischaemia or heart failure, virilisation in females.

ETHICS AND DISSEMINATION

Research ethics approval

All research ethical and regulatory approvals will be sought and obtained at each site from the responsible local and/or national human research ethics committee in accordance with National and State Requirements prior to the start of the study The study has been designed in a manner to ensure that the welfare of participants is protected at all times and that respect for their autonomy is demonstrated through appropriate mechanisms of consent.

Protocol amendments

Protocol amendments will be updated on relevant clinical trial registries by the Project Manager. Amendments will be communicated by regular newsletters, teleconferences, and emails to site Principal Investigators and Research Co-ordinators.

Consent

The majority of patients enrolled in this trial will have capacity to give consent at the time of trial enrolment. The following consent options are acceptable: (i) consent by the patient (ii) consent by a substitute decision maker using the IMP / GAA pathway for WA Health; (iii) Delayed consent from the patient. All participants who recover sufficiently will be given the opportunity to provide informed consent for ongoing study participation and for the use of data collected for the study.

Confidentiality

Patients will be allocated a unique study number. The site research co-ordinator will compile an enrolment log that includes the patients' details and a unique study number. Study data will be obtained from the patients' medical records. The study data and the study enrolment logs will be kept separately. Contact details for participants and their next of kin will be provided to the Project Manager and quality of life outcome data will be obtained by a research coordinator by phoning participants and/or their next of kin.

Declaration of interests

All study investigators have confirmed that they do not have any financial or other conflicts of interest to declare in relation to this study.

Access to data

The final trial dataset will be available to the study investigators. There are no contractual agreements in place which limit access to study data.

Dissemination policy

The trial will be conducted in the name of the GAINS Investigators.
The principal publications from the trial will be in the name of the GAINS Investigators with full credit assigned to all collaborating investigators, research coordinators and institutions. Where individuals' names are required for publication they will be the members of the management committee, with the chair of the writing committee listed first and subsequent authors listed alphabetically. Funding bodies will be acknowledged in the publication.

2 REFERENCES

1. Dinglas VD, Friedman LA, Colantuoni E, Mendez-Tellez PA, Shanholtz CB, Ciesla ND, et al. Muscle Weakness and 5-Year Survival in Acute Respiratory Distress Syndrome Survivors. Crit Care Med. 2017 Mar;45(3):446–53.

2. Herridge MS, Tansey CM, Matté A, Tomlinson G, Diaz-Granados N, Cooper A, et al. Functional disability 5 years after acute respiratory distress syndrome. New England Journal of Medicine. 2011;364(14):1293–304.

3. Nierman DM, Mechanick JI. Hypotestosteronemia in chronically critically ill men. Crit Care Med. 1999 Nov;27(11):2418–21.

4. Almoosa KF, Gupta A, Pedroza C, Watts NB. Low Testosterone Levels are Frequent in Patients with Acute Respiratory Failure and are Associated with Poor Outcomes. Endocr Pract. 2014 Oct;20(10):1057–63.

5. Anstey MH, Rauniyar R, Fitzclarence E, Tran N, Osnain E, Mammana B, et al. The muscle Growth and Anabolism in Intensive Care Survivors (GAINS) trial: a pilot randomised controlled trial. Acute and Critical Care. 2022;

6. Norman R, Viney R, Brazier J, Burgess L, Cronin P, King M, et al. Valuing SF-6D health states using a discrete choice experiment. Medical Decision Making. 2013;0272989X13503499.
