# Supplementary material for: Muscle growth and anabolism in intensive care survivors (GAINS 2.0): Protocol for a multi-centre randomised; placebo controlled clinical trial of nandrolone in deconditioned adults recovering from critical illness
Source: PLoS One. 2025 Feb 20;20(2):e0315170. doi: 10.1371/journal.pone.0315170 (PMC11841879; doi:10.1371/journal.pone.0315170)
Supplement: Attachment — Submitted filename: Reply reviewers.docx [file pone.0315170.s003.docx]

Thank you to the Editor and Reviewers for your thoughtful comments on the trial paper. It was helpful to refine the paper.

Our responses are outlined below.

**Reviewer #1:**

*Blinding Procedure: The blinding procedure must be more clearly described. It should be explicitly stated how placebo formulations and preparations are done in the study - what is used as placebo, what volume etc.. The details on how blinding will be maintained throughout the study should be expanded - i.e. that there are no information rute from the person preparing the study drug to the one administering the drug.*

Thank you. The paper states “Placebo is defined as sterile water or normal saline injection” and we have added “to the same volume as the drug (1 or 2mL)”

The paper also states “The study drug will be drawn up by a blinded member of the investigational team not otherwise involved in the care of the participant (e.g pharmacist or senior nurse) with the syringe covered (to mask the contents of the syringe).” – ie the person administering is blinded to the preparation.

*Randomization: The current randomization method involves the use of sealed envelopes. This approach is considered outdated compared to more modern solutions such as computer-generated randomization. The study should justify the choice of sealed envelopes. Clarify if any stratification or block randomization will be implemented to ensure balance among different patient groups.*

Thank you for noticing this. Indeed we have evolved this process to use a computerised system - the randomization uses randomization embedded within the RedCap system, stratified by site.

*Inclusion Criteria: The inclusion criteria need to be more specific. It is important to define the exact conditions under which patients will be assessed for inclusion. The current criteria suggest that any sedated or otherwise immobilized patient could be included. Clear guidelines should be established to determine which patients are eligible for the study to avoid ambiguity.*

Thank you – we have added the clarification that the inclusion criteria require the patient to be able to interact with rehabilitation, which means they can’t be sedated or immobilised.

*Power Calculation: The power calculation should be extended to include assumptions regarding the distribution of the outcome, supported by the best available data. The current mention of proportions in relation to a time-to-event outcome is unclear and needs clarification. Length of Stay (LOS) is a challenging outcome due to mortality bias. The study should justify why LOS was chosen over more recognized metrics such as days alive and out of hospital (DAOH) as the primary outcome.*

*Statistical Significance Level: The statistical significance level should be explicitly reported. Additionally, a power calculation for secondary outcomes should be provided to ensure that the study is adequately powered to detect differences in these outcomes as well.*
*Statistical Analysis: As this is a randomized study, it is generally discouraged to adjust for confounding factors. The study should consider not adjusting for these factors to maintain the integrity of the randomization process and avoid introducing potential biases.*

Thank you. In discussion with our statistician (now added to the paper as a co-author), we have adjusted the statistical analysis section.

The key change is that instead of length of hospital stay, we will report “number of days out of hospital since enrolment out of 90 days”.

The text now reads

Power calculations for primary outcomes MRC (continuous), IMS>8 (binary) and number of days out of hospital since enrolment out of 90 days (count) and secondary outcome grip strength follow. A sample of n=46 has 80% power (alpha=0.05) to detect a mean±SD difference of 7±10 in MRC and grip strength (effect size difference f=0.35) between 2 groups over a minimum of 3 timepoints in a repeated measures ANOVA between factors (mixed) model. A sample of n=46 has 80% power (alpha=0.05) to detect an odds ratio of 2.75 in a logistic model, based on a group proportional difference of 24.5% for IMS>8 (pilot study indicated a 27.2% proportional difference). Based on a Poisson regression for count data, a sample of n=44 has 80% power to detect a group difference in number of days out of hospital at 90 days from enrolment of 7 (70 vs 63 days). Allowing for 25% loss to follow-up, a total of 60 patients will be recruited. G*Power 3.1.9.7 (University of Dusseldorp and PASS Sample Size (NCSS, LLC) was used for power calculations.

**Reviewer #2:** 

*There are only 60 patients to be recruited. The randomisation is stratified by site - which is okay so long as blinding is sufficiently good, but given the different dose by sex, stratification by sex is important too.*

*The sample size calculation is entirely inadequate here. Please give the actual figures used to derive a total number of patients.*

Updated.

*The analyst cannot be truly blind to assignment - this needs to be reworded.*

*How are people who do not leave hospital - or die in hospital - allowed for in the length of stay analysis. Length of stay is unlikely to be normally distributed. There are unlikely to be enough patients and events to allow for the amount of adjustment mentioned here.*

Thank you. As discussed above, the outcome will now be days out of hospital at 90 days from enrolment.

*The people referenced in the text are Student (capital S) and R A Fisher (not Fischer). Why are both logrank tests and Cox regression being used for unadjusted analyses? Which RR is going to be the primary analysis?*

The statistical analysis section has been updated.

The analysis will be by intention to treat population, defined as all study participants except for those who do not consent to the use of data.

Descriptive summaries of patient and outcome data will consist of means and standard deviations or medians and interquartile ranges for continuous data and frequency distributions for categorical data. Univariate group comparisons between treatment arms will be done using t-tests or Mann-Whitney U tests for continuous data, depending on normality of distributions, and Chi-squared or Fisher’s Exact tests for categorical data comparisons.

Generalised linear mixed models (GLMM) will be used to examine longitudinal MRC and grip strength with results summarised as estimated means and mean differences with 95% confidence intervals (CI). Interaction effects (difference of the group differences over time) will be reported. Mixed effects logistic models will be used to examine the odds of walking independently (IMS>8) with results summarised as odds ratios (OR) and 95% CI.

Poisson regression will be used to compare number of days out of hospital at 90 days and days to return to work from enrolment between groups, with results summarised as estimated means and mean differences with 95% CI.

Linear regression models will be used to compare SF36 quality of life subscale outcomes between groups, with results summarised as estimated means and mean differences with 95% CI.

All mixed effects models will incorporate random subject effects and group-time interactions and all models will include covariates ICU length of stay, age, gender, BMI, APACHE 2 score and baseline MRC.

Times to mortality and morbidity events will be described using Kaplan-Meier probabilities and compared between groups using log-rank tests. Results will be summarised using medians and 95% CI. Cox proportional hazards regression models may be used to describe adjusted hazard ratios (HR) and 95% CI where univariate differences are shown.

Significance will be set at alpha=0.05. Stata version 18 (StataCorp, College Station, TX) will be used for data analysis.

*Has the acceptability of a dummy IM injection - which comes with issues of discomfort etc - been piloted within the hospital population of interest?*

I assume the reviewer is suggesting that the IM placebo may be painful and therefore distressing to participants. We have not had any complaints when we have conducted the pilot study previously about this, and to help maintain the integrity of blinding of clinical staff, we think it is important to have the placebo arm in the trial.
