# Supplementary material for: Muscle growth and anabolism in intensive care survivors (GAINS 2.0): Protocol for a multi-centre randomised; placebo controlled clinical trial of nandrolone in deconditioned adults recovering from critical illness
Source: PLoS One. 2025 Feb 20;20(2):e0315170. doi: 10.1371/journal.pone.0315170 (PMC11841879; doi:10.1371/journal.pone.0315170)
Supplement: Attachment — Submitted filename: Reply reviewers_2.docx [file pone.0315170.s004.docx]

Thank you to the Editor and Reviewers for your thoughtful comments on the trial paper. It was helpful to refine the paper.

Our responses are outlined below.

**Reviewer #1:**

*It is unclear whether the adjusted mixed effects models are intended as the primary or secondary/sensitivity analysis. If they are intended as the primary analysis, this should be strongly justified and factored into the sample size calculation. There are 6 variables for 60 patients, which may lead to an inflation of statistical power. In general, adjustment is not recommended in the primary analysis of randomized trials.*

Thank you, primary outcome models will not be adjusted.

*The "days out of hospital" outcome is not mentioned or defined in the outcomes section. Please also explain how patients who die during follow-up are handled. How is missing follow-up data addressed?*

Days out of hospital will be modelled using survival methods if date of death is available,censoring on post-discharge death. If date of death not available ,number of days for non-survivors set to zero.

*In the sample size section, the authors justify needing approximately 45 patients but round this number up to 60 due to an expected 25% loss to follow-up. A 25% expected loss to follow-up is a significant number that could skew the results. The authors must justify this estimate and explain how it will be addressed, including any potential bias it may introduce. If the 25% figure is more of a convenience decision to reach a sample size of 60, please consider revising to only include the number necessary to avoid exposing additional patients to the intervention unnecessarily.*

Thank you. In using 25%, we considered the patient cohort, ie those recovering from severe critical illness, and that is what we originally based the 25% loss to follow-up on. We have re-looked at our pilot study, which had 2 deaths out of 22 patients – so we have revised the sample size figure to a 15% loss to follow up = 54 patients.

*Given the complexity of the described analysis, it is recommended to complete a separate, full statistical analysis plan before unblinding the data.*

A statistical analysis plan will be prepared and submitted to IDMC if required, for approval prior to unblinding.

**Reviewer #2:**

*Thank you for your response to my previous concerns - I do not see a data sharing protocol here and it should be in the data management section here.*

Thank you. In the PLOSONE template we have the following statement : No datasets were generated or analysed during the current study.

We have added this to the manuscript:

No datasets were generated or analysed during the current study. De-identified data can be made available at the request of the investigators and the approval of the administering ethics organisation at the completion of the study.
